# Efficacy of Intermittent Theta‐Burst Stimulation for Prolonged Disorders of Consciousness: A Prospective, Randomized, Controlled Trial

**DOI:** 10.1002/acn3.70342

**Published:** 2026-02-19

**Authors:** Chuan Xu, Jun Hu, Qianqian Wu, Min Wu, Yamei Yu, Hangcheng Li, Jian Gao, Jingqi Li, Nai Ding, Jie Yu, Benyan Luo

**Affiliations:** ^1^ Department of Neurology Sir Run Run Shaw Hospital, School of Medicine, Zhejiang University Hangzhou China; ^2^ Department of Neurology First Affiliated Hospital, School of Medicine, Zhejiang University Hangzhou China; ^3^ Sir Run Run Shaw Hospital, School of Medicine, Zhejiang University Hangzhou China; ^4^ Department of Rehabilitation Hangzhou Mingzhou Brain Rehabilitation Hospital Hangzhou China; ^5^ Key Laboratory for Biomedical Engineering of Ministry of Education, College of Biomedical Engineering and Instrument Sciences Zhejiang University Hangzhou China

**Keywords:** disorders of consciousness, iTBS, low‐frequency oscillations, prognosis

## Abstract

**Background:**

Emerging evidence suggests that low‐frequency neural oscillations are dynamically regulated by consciousness levels, with the recovery of low cortical activity potentially serving as a neurophysiological substrate for conscious emergence. Targeted enhancement of these low‐frequency rhythms in patients with disorders of consciousness (DoC) may constitute a promising neuromodulation strategy to facilitate consciousness recovery in severe brain injury.

**Objective:**

This study systematically examined the neurophysiological effects of intermittent theta‐burst stimulation (iTBS), specifically its potential to enhance low‐frequency cortical activity and promote consciousness recovery in patients with DoC. Through multimodal neural assessments, we aimed to elucidate the mechanistic relationship between iTBS‐induced neural oscillation modulation and behavioral manifestations of consciousness improvement.

**Results:**

This prospective cohort study enrolled 30 patients with DoC, of whom 18 completed the full intervention protocol. Two‐way repeated‐measures analysis of variance revealed significant group × time interaction effects on the Coma Recovery Scale‐Revised (CRS‐R) scores, *F*(1, 16) = 6.543, *p* = 0.021. Post hoc simple effects analysis demonstrated significant temporal improvement in the active transcranial magnetic stimulation (TMS) group, *F*(1, 16) = 36.463, *p* < 0.001, with mean CRS‐R scores increased from 9.300 ± 1.320 at baseline to 11.700 ± 1.409 post‐intervention (*p* < 0.001). Conversely, sham stimulation revealed statistically nonsignificant changes (9.845 ± 1.476 versus 10.750 ± 1.575, *p* = 0.067). Neurophysiological assessments revealed emerging neurophysiological changes in the iTBS group, including enhanced resting‐state low‐frequency oscillations (delta: 21.642% increase, *p* = 0.449; theta: 6.800% increase, *p* = 0.789) and augmented auditory‐evoked responses (phrase‐level 22.917% increase, *p* = 0.280; syllable‐level: 22.963% increase, *p* = 0.504), suggesting potential neural plasticity mechanisms that require further validation.

**Conclusion:**

Collectively, this study established iTBS targeting the left dorsolateral prefrontal cortex as a clinically effective and well‐tolerated neuromodulation approach for consciousness rehabilitation in patients with DoC, with therapeutic effects mediated by iTBS‐induced enhancement of thalamocortical low‐frequency oscillations.

**Trial Registration:**

https://www.clinicaltrials.gov. Unique identifier: NCT03385278. Registered on October 24, 2017

## 1 Introduction

Slow cortical activity is considered optimal for conducting large‐scale information integration in the brain, contributing to the emergence of consciousness and attention [1, 2]. As an essential part of slow cortex activity, theta oscillation is generally hypothesized to be functionally relevant for cognitive processing, including spatial navigation and working memory [3]. Theta oscillation reflects thalamocortical connectivity, and the improvement of consciousness in patients with disorders of consciousness (DoC) is indicated by the emergence of theta oscillations [4, 5]. Therefore, theta oscillation may be an effective intervention target for consciousness‐promoting awakening in severe brain injury, including patients with DoC. DoC includes coma (unwakefulness and reflex behaviors only), unresponsive wakefulness syndrome (UWS)/previously known as vegetative state (VS), and minimally conscious state (MCS) [6]. In patients with VS and MCS, their arousal ability is the same; however, their awareness ability is different. Although it has been suggested that some patients with prolonged DoC may benefit from therapeutic intervention [7, 8, 9], even years after the injury, the available treatment options for prevention remain scarce [10]. Consequently, it is vital to develop alternative therapeutic approaches to treat DoC.

Intermittent theta‐burst stimulation (iTBS) is a novel repetitive transcranial magnetic stimulation (TMS) [rTMS] protocol that mimics endogenous theta rhythm. To improve the excitability and plasticity of the cerebral cortex, it may be reasonable to use iTBS to directly entrain theta oscillations. iTBS has been proposed as a promising therapeutic intervention for unconscious disorders. iTBS increases brain activity in patients in a vegetative state [11]. In a case report, iTBS improves the consciousness of patients with DoC, and theta power in patients with DoC tended to increase after iTBS intervention [12]. However, iTBS has not yet revealed significant improvements in consciousness among the DoC population. Moreover, the neural rehabilitation mechanism of iTBS for consciousness improvement in patients with DoC remains unclear.

This study aimed to evaluate the iTBS efficacy in awakening consciousness in patients with DoC using a prospective, randomized, controlled trial. A comprehensive clinical and electrophysiological evaluation, including behavioral assessment via CRS‐R, intrinsic and auditory‐evoked neural oscillations, was performed to determine low cortical activity enhancements following iTBS and the improvement in consciousness in patients with DoC.

## 2 Methods

### 2.1 Study Design and Participants

This prospective, randomized, controlled trial (ClinicalTrials.gov ID: NCT03385278) used a computer‐generated randomization protocol to allocate patients with DoC into active iTBS (*n* = 15) or sham‐controlled (*n* = 15) groups at Hangzhou Mingzhou Brain Rehabilitation Hospital. The 14‐day intervention protocol comprised daily 600‐pulse iTBS sessions (50 Hz triplets, 5 Hz burst frequency) delivered to the left dorsolateral prefrontal cortex (DLPFC); however, sham stimulation used identical auditory cues with a 90° coil tilt. CRS‐R scores, resting state, and auditory‐evoked electroencephalography (EEG) were performed at the beginning (T0) and post‐intervention (T1) after a sufficient course of intervention (iTBS or sham intervention). The inclusion criteria were as follows: (i) MCS or UWS diagnosis via CRS‐R assessments by DoC experts [6]; (ii) over 1 month post‐brain injury; (iii) age above 18; (iv) no pre‐injury hearing impairment; (v) preserved auditory startle reflex; (vi) absence of centrally acting drugs, neuromuscular blockers, or sedation 24 h before the study; (vii) nonsignificant skull bone defects (CT); (viii) CT‐confirmed brain lesions not restricted to the brainstem, excluding locked‐in syndrome. Patients experienced traumatic brain injury, anoxic brain injury, or cerebrovascular disease. The study received approval from the Ethics Committees of the Hangzhou Mingzhou Brain Rehabilitation Hospital. All patients' guardians provided written informed consent for the experiments. All clinical work was conducted following the principles of the Declaration of Helsinki.

### 2.2 Study Interventions: iTBS and Sham Stimulation

Target localization was located on the left DLPFC, identified through a position cap designed following the 10–20 electrode system. Each patient received five sessions of active or sham iTBS a week for 2 weeks (one session of active or sham iTBS/day, consecutive stimulation for 5 days and 2 days off, 10 sessions received for each patient). The iTBS pattern settings were as follows: Three pulses, 50 Hz bursts given every 200 ms (at 5 Hz), and an intensity of 70% of the resting motor threshold. The iTBS cycles were delivered in 2‐s trains, with an 8‐s inter‐train interval and a duration of 3 min and 20 s. Consequently, each patient received 600 pulses per day (total: 600 pulses/day × 10 days). In the sham group, the parameters were the same as in the iTBS group, and the stimulation coil was tilted 90° relative to the north of the skull [13]. Therefore, patients with DoC in the sham group did not receive effective TMS stimulation.

### 2.3 Multimodal Assessment Protocol

Primary outcomes included the following:

Behavioral evaluation: CRS‐R administered at baseline (T0) and post‐intervention (T1).

Neurophysiological monitoring: Resting‐state EEG (64‐channel brain products EEG system) and auditory‐evoked EEG (see later).

### 2.4 Procedures

According to the computer‐generated list, patients with DoC were randomized into either iTBS or sham groups. Physicians and nurses were aware of the group assignments. The principal investigators enrolled participants and assigned them to the intervention. Before accepting iTBS or sham stimulation, all participants received standard behavioral assessments (CRS‐R scores), resting‐state, and auditory‐evoked EEG recordings (T0). A 64‐electrode BrainCap (Brain Products DmbH, Munich, Germany) was used to record the EEG data. The resting‐state EEG duration was at least 5 min. Regarding auditory‐evoked EEG, all participants passively listened to isochronous speech sequences while their EEG responses were being recorded. The auditory sequence was the same as that in Experiment 2 of Xu et al. [14]. The syllables constructed four trisyllabic artificial words that were adapted from the seminal study by Saffran et al. [15]. The four trisyllabic artificial words were “bīdākū”, “goūlābū”, “tūpīroū”, and “pādoūtī”. After completing the behavioral and EEG assessments, all patients received either iTBS or sham stimulation, following the patient grouping list. After a sufficient course of intervention, all subjects underwent behavioral evaluation, resting‐state, and auditory‐evoked EEG recording again (T1).

### 2.5 EEG Recording and Preprocessing

EEG signals were captured using a 64‐electrode BrainCap (Brain Products DmbH, Munich, Germany), including an electrode positioned beneath the right eye for electrooculogram (EOG) recording. EEG signals were initially referenced to FCz online and later adjusted offline to the average of bilateral mastoids. The EEG signals were processed using a 12th‐order zero‐phase Butterworth notch filter at 50 Hz to eliminate line noise, an 8th‐order zero‐phase Butterworth low‐pass filter with a 70 Hz cutoff for anti‐aliasing, and an 8th‐order zero‐phase Butterworth high‐pass filter with a 0.3 Hz cutoff to mitigate slow drifts. The signals were sampled at 1 kHz and downsampled to 200 Hz. The least‐squares method was used to regress the EOG artifacts. The EEG signal was processed following the method outlined by Zou et al. [16]. The preprocessing and analyses of this study were conducted using MATLAB software (The MathWorks, Natick, MA).

### 2.6 Frequency‐Domain Analysis

Each condition's trial consisted of a 10‐s EEG data segment. Therefore, each condition consisted of 108 trials. A Discrete Fourier Transform (DFT) was applied to the 10‐s data segment. The inter‐trial phase coherence (ITPC) is defined as follows:

$$C_{f}={\left(\sum_{t=1}^{T}\cos\left(\alpha_{\text{ft}}\right)\right)}^{2}+{\left(\sum_{t=1}^{T}\sin\left(\alpha_{\text{ft}}\right)\right)}^{2}$$

where *α*_*ft*_ represents the DFT phase at frequency *f*, and *C*_*f*_ signifies the ITPC. An elevated ITPC value signifies consistent response phases across the trials.

The EEG amplitude spectrum was generated using the Discrete Fourier transform. At each channel, the amplitude within two canonical frequency bands, delta (0–4 Hz) and theta (4–8 Hz), was calculated.

### 2.7 Statistical Analysis

Treatment outcomes were examined using a two‐factor repeated‐measures analysis of variance. If there was no interaction between group (iTBS and sham) and time (baseline and week 2), we focused on analyzing the main effect. If there was an interaction, we focused on analyzing the interaction effect and the simple main effect.

## 3 Results

**FIGURE 1:**
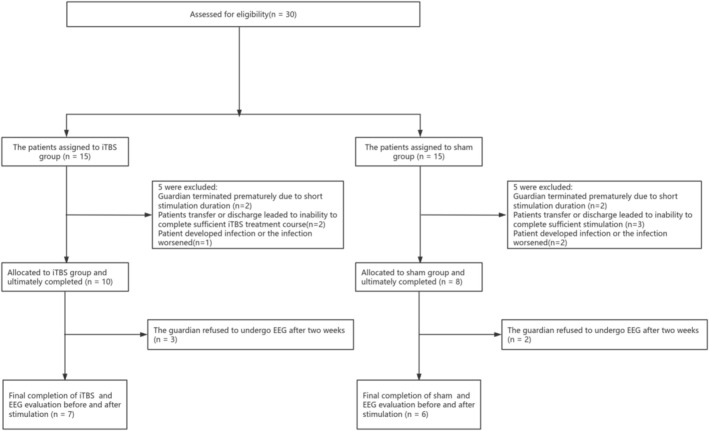
Flowchart of iTBS for patients with DoC. DoC, disorders of consciousness; iTBS, intermittent theta‐burst stimulation.

**FIGURE 2:**
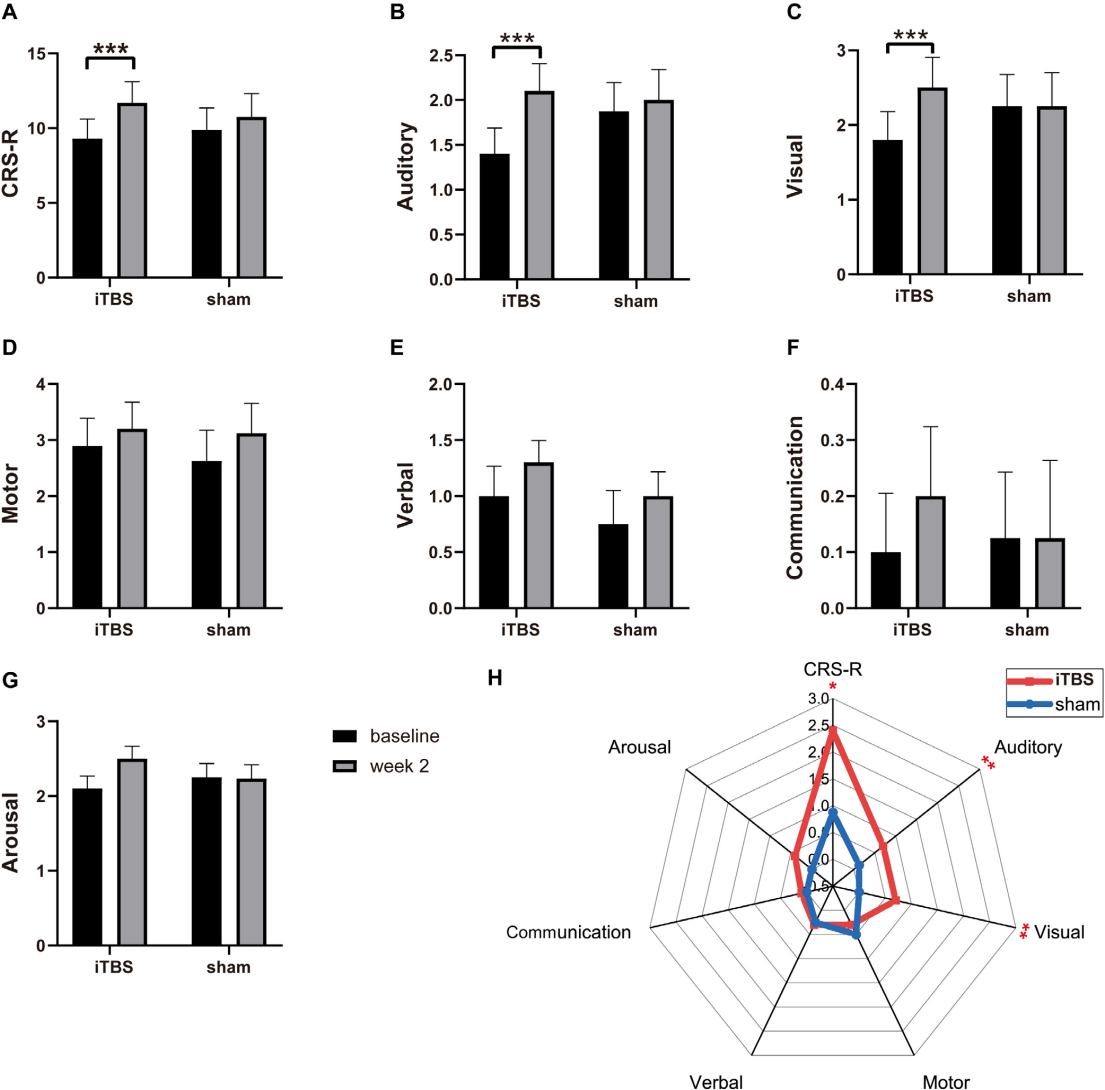
After effects of iTBS treatment in active and sham groups. (A) Improvement of the CRS‐R scores after intervention; (B) Improvement of the auditory subscores after intervention; (C) Improvement of the visual subscores after intervention; (D) Improvement of the motor subscores after intervention; (E) Improvement of the verbal subscores after intervention; (F) Improvement of the communicaiton subscores after intervention; (G) Improvement of the arousal subscores after intervention; (H) The symptom change after intervention at week 2. Red stars indicate the interaction effects of group (iTBS and sham) by time (baseline and week 2). *p < 0.05; **p < 0.01; ***p < 0.001.

Initially, 30 patients with DoC were selected for inclusion in this study and randomized to active iTBS (*n* = 15) or sham‐controlled (*n* = 15) groups using a computer‐generated randomization protocol. Ten patients in the active iTBS group and eight patients in the sham group completed the clinical trial after a sufficient course of intervention (iTBS or sham intervention). There were nonsignificant differences in baseline demographic information, including age, sex, etiological distribution, and CRS‐R score from baseline between the two groups of patients (Table 1). Seven patients in the active iTBS group and six in the sham group completed the final neurophysiological evaluation (Figure 1). There was a significant main effect of time (baseline and week 2) on CRS‐R scores, *F*(1, 16) = 30.177, *p* < 0.001, and a nonsignificant main effect of group (iTBS and sham) on CRS‐R scores, *F*(1, 16) = 0.009, *p* = 0.927. Furthermore, interaction effects of group (iTBS and sham) by time (baseline and week 2) were observed in CRS‐R scores, *F*(1, 16) = 6.543, *p* = 0.021, indicating that the improvement in consciousness in the iTBS treatment group was greater than that in the sham treatment group over time. The simple main effect was then analyzed. In the iTBS group, the simple effect of time was significant, *F*(1, 16) = 36.463, *p* < 0.001. In the sham group, the simple effect of time was nonsignificant, *F*(1, 16) = 3.877, *p* = 0.067. Post hoc comparisons were performed to test the differences between time points (baseline and week 2) in the iTBS or sham group. As revealed in Figure 2A, the CRS‐R score at week 2 was significantly greater on baseline in the iTBS group; however, not in the sham group. These results indicate that TMS (iTBS pattern) can improve CRS‐R scores in patients with DoC.

**TABLE 1:** Baseline characteristics of DoC patients with iTBS or sham.

	iTBS	Sham	
DoC	10	8	
MCS	7	6	
UWS	3	2	
Age (years)	54.60 ± 5.13	66.75 ± 2.13	0.056
Sex (M/F)	6/4	7/1	0.196
Etiology			
TBI (*n*)	4	4	0.671
nTBI (*n*)	6	4	
CRS‐R (T0)	9.30 ± 1.23	9.88 ± 1.48	0.775

Abbreviations: CRS‐R, Coma Recovery Scale‐Revised; MCS, minimally conscious state; nTBI, nontraumatic brain injury; TBI, traumatic brain injury; UWS, unresponsive wakefulness syndrome.

The effect of iTBS on CRS‐R subscores was then analyzed. There was a significant main effect of time (baseline and week 2) on auditory and visual scores, *F*(1, 16) = 16.269, *p* = 0.001; *F*(1, 16) = 16.593, *p* = 0.001, and a nonsignificant main effect of group (iTBS and sham) on auditory and visual scores, *F*(1, 16) = 0.187, *p* = 0.671; *F*(1, 16) = 0.187, *p* = 0.671. Furthermore, group (iTBS and sham) by time (baseline and week 2) interaction effects were observed in the auditory and visual scores, *F*(1, 16) = 7.903, *p* = 0.013; *F*(1, 16) = 16.593, *p* = 0.001. In the iTBS group, the simple main effect of time was significant in auditory and visual scores, *F*(1, 16) = 26.353, *p* < 0.001; *F*(1, 16) = 37.333, *p* < 0.001. In the sham group, the simple effect of time was nonsignificant in auditory and visual scores, *F*(1, 16) = 0.672, *p* = 0.424; *F*(1, 16) = 0.000, *p* = 1.000. As revealed in Figure 2B,C, post hoc comparisons between times (baseline and week 2) indicated that the auditory and visual scores on week 2 were significantly greater than baseline in the iTBS group; however, not in the sham group. For motor and verbal scores, there were significant main effects of time, *F*(1, 16) = 5.619, *p* = 0.031; *F*(1, 16) = 5.975, *p* = 0.026, respectively and nonsignificant main effects of group, *F*(1, 16) = 0.061, *p* = 0.808; *F*(1, 16) = 0.681, *p* = 0.421, respectively and group (iTBS and sham) by time (baseline and week 2) interaction effects, *F*(1, 16) = 3.351, *p* = 0.562; *F*(1, 16) = 0.049, *p* = 0.827, respectively. There were nonsignificant main effects of time, group and group (iTBS and sham) by time (baseline and week 2) interaction effects in communication scores, *F*(1, 16) = 0.790, *p* = 0.387; *F*(1, 16) = 0.023, *p* = 0.880; *F*(1, 16) = 0.790, *p* = 0.387, respectively and in arousal scores, *F*(1, 16) = 3.716, *p* = 0.072; *F*(1, 16) = 0.004, *p* = 0.953; *F*(1, 16) = 1.019, *p* = 0.328, respectively. The Group (iTBS and sham) by time (baseline and week 2) interaction effects in the CRS‐R, auditory, and visual scores are intuitively revealed in Figure 2H, and most tests revealed improved scores in the iTBS group than in the sham group after treatment.

**FIGURE 3:**
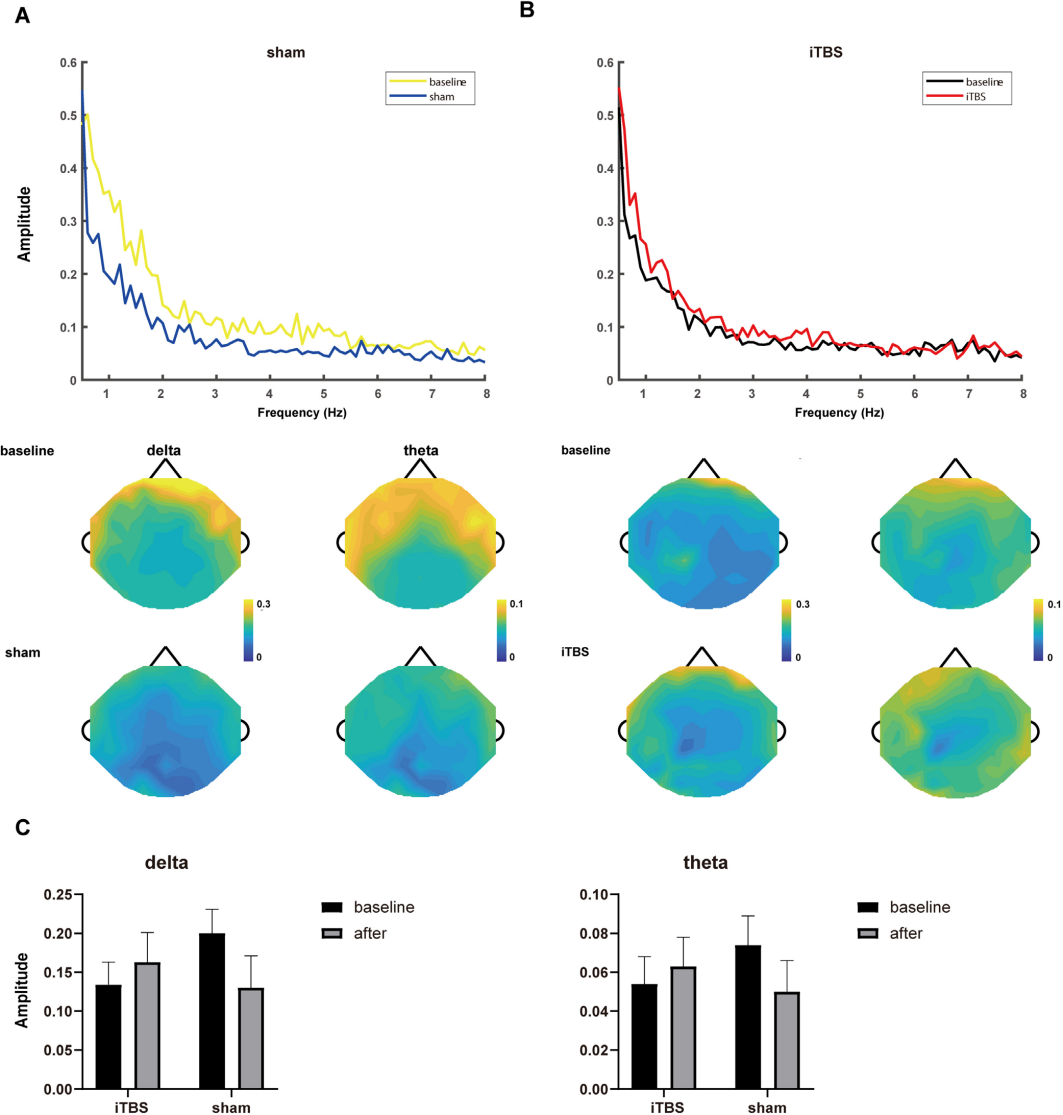
Rest EEG spectrum and topography. (A) Rest EEG spectrum and topography in the TMS group (iTBS pattern); (B) Rest EEG spectrum and topography in the sham group. (C) The delta and theta amplitude change in iTBS and sham groups (mean ± standard error of the mean).

The rest of the EEG was recorded to analyze the effect of iTBS stimulation on brain electrical activity. As presented in Figure 3, an EEG spectrum analysis indicated that there were nonsignificant main effects of time, group and group (iTBS and sham) by time (baseline and week 2) interaction effects in delta (*p* = 0.478; *p* = 0.692, *p* = 0.099, respectively) and theta amplitudes (*p* = 0.440; *p* = 0.959; *p* = 0.271 respectively). There was a decreasing trend in delta and theta amplitudes in the sham group after 2 weeks, with delta amplitude decreasing from 0.200 ± 0.031 at baseline to 0.130 ± 0.041, with theta amplitude decreasing from 0.074 ± 0.015 at baseline to 0.050 ± 0.016 sham post‐intervention, whereas an increasing trend in delta and theta amplitudes in the TMS group after 2 weeks, with delta amplitude increasing from 0.134 ± 0.029 at baseline to 0.163 ± 0.038, with theta amplitude increasing from 0.054 ± 0.014 at baseline to 0.063 ± 0.015 iTBS post‐intervention. The scalp topography in delta and theta amplitude indicated that delta and theta amplitudes in the whole brain area were enhanced after iTBS treatment.

**FIGURE 4:**
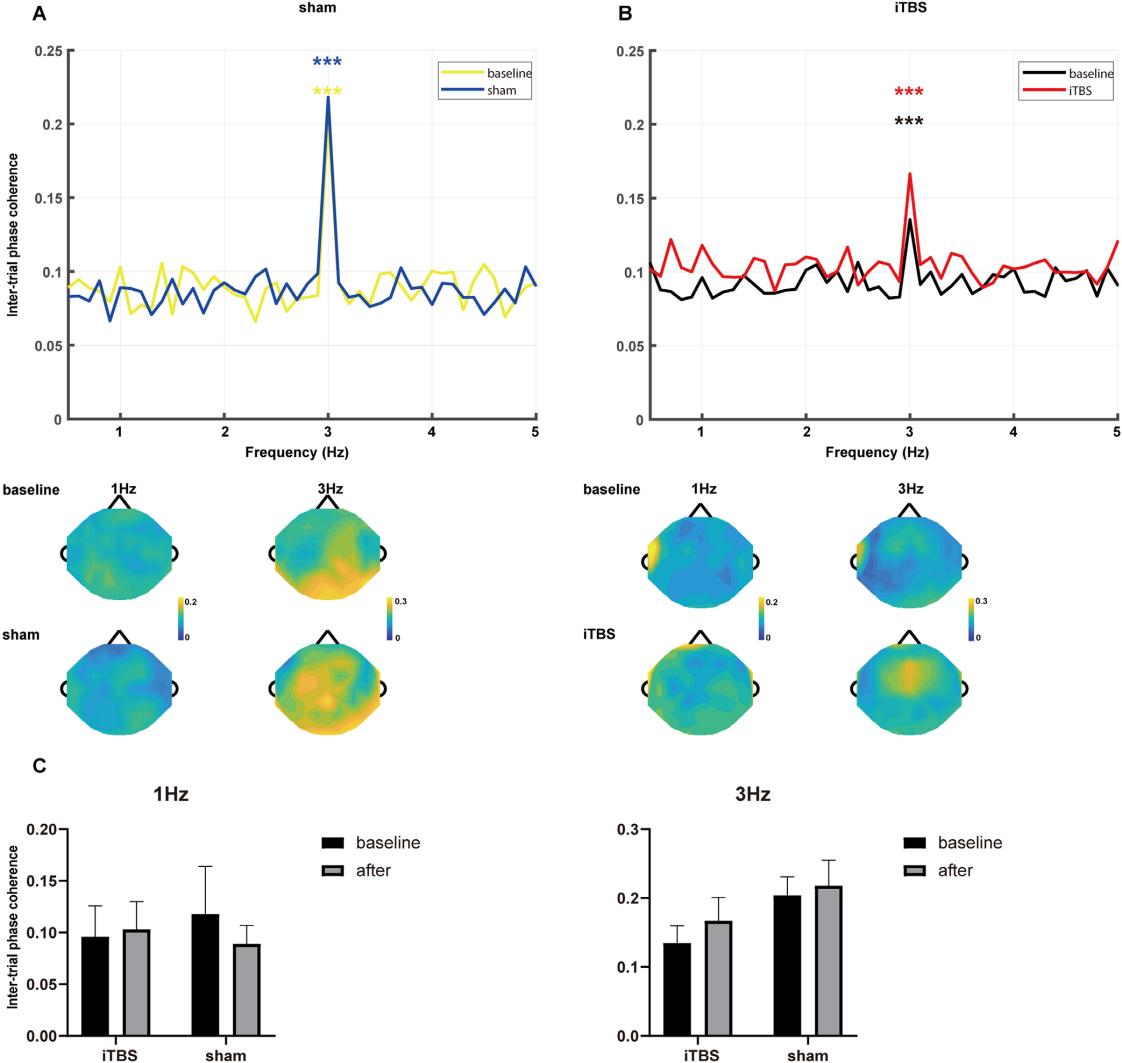
EEG response spectrum and topography. (A) The iTPC at target frequencies for the sham group; (B) The iTPC at target frequencies for the TMS group; (C) The iTPC change at target frequencies for sham and iTBS groups.

The EEG responses to auditory stimuli are revealed in Figure 4. There were nonsignificant main effects of time and groups (iTBS and sham) by time (baseline and week 2) interaction effects in syllable‐level neural tracking (3‐Hz ITPC) (*p* = 0.511; *p* = 0.063; *p* = 0.800 respectively) and phrase‐level tracking (1‐Hz ITPC) (*p* = 0.784; *p* = 0.348; *p* = 0.233 respectively). There was a decreasing trend in phrase‐level tracking in the sham group after 2 weeks, whereas a trend of increasing phrase‐level tracking was observed in the iTBS group after 2 weeks. The scalp topography in phrase‐level tracking indicated that neural tracking to phrase in a centro‐frontal region was enhanced after iTBS treatment.

## 4 Discussion

In this study, iTBS was used to improve consciousness in patients with DoC. iTBS significantly improved the CRS‐R scores in patients with DoC, whereas sham stimulation did not. Moreover, intrinsic and auditory‐evoked theta oscillations revealed an increasing trend after iTBS stimulation. These results suggested iTBS had the potential to improve consciousness in patients with DoC by improving theta oscillation.

Theta burst stimulation (TBS) is a novel form of rTMS that mimics the natural brain activity during learning tasks [17]. iTBS is an effective method to induce long‐term potentiation (LTP)‐like plasticity in humans by enhancing synaptic function [18]. Therefore, iTBS is considered excitatory for brain activity and is a noninvasive brain stimulation technology used to modulate the target neural networks underlying psychiatric and neurological disorders [19, 20]. iTBS stimulates the supplementary motor area to improve motor sequence learning [21]. Moreover, iTBS improves treatment‐resistant depression and poststroke motor deficits [22, 23]. Regarding DoC, it is revealed that iTBS can alter the oscillation power, complexity, and functional connectivity of the brain activity of patients with VS [11]. In the behavioral manifestations of DoC, iTBS has not yet revealed an improvement in awareness of prolonged DoC. Only one case report indicates that iTBS has a consciousness‐improving effect on some patients with DoC [12]. In this study, a double‐blind, randomized clinical trial was designed to determine the improvement of iTBS on consciousness in patients with DoC, and it was revealed that iTBS of the left DLPFC had an effective and well‐tolerated treatment for patients with DoC.

iTBS is a new pattern of rTMS and has been approved to treat treatment‐resistant depression [24]. Accelerated iTBS of the DLPFC is an effective and well‐tolerated complementary treatment for patients with AD, specifically for individuals with relatively high MMSE scores [25]. In patients with DoC, 20 Hz rTMS has been revealed to have therapeutic efficacy in improving consciousness in some patients with DoC, possibly because high‐frequency rTMS causes long‐term potentiation [26]. In a case report, it is revealed that iTBS of the left dorsolateral prefrontal cortex improves consciousness in most patients with DoC [12]. However, this study did not include a control group. In a sham‐controlled crossover study, one session of iTBS significantly altered the brain activity of patients with VS and has the potential to improve consciousness in patients with DoC [11]. In a prospective, randomized, controlled trial, the CRS‐R scores in patients with DoC were significantly improved in the iTBS group; however, not in the sham group. Moreover, intrinsic and auditory‐evoked theta oscillations exhibited an increasing trend after iTBS stimulation. iTBS mimics endogenous theta rhythms in the brain. Theta oscillations reflect cognitive processing during tasks and have been identified as indicators of intense cognition [27]. Therefore, theta oscillation is a key target mediating cognitive function. In this study, the rehabilitation mechanism by which iTBS exerts a consciousness‐improving effect may be the enhancement of theta oscillations.

Besides endogenous theta oscillations, auditory‐evoked theta oscillations have also been enhanced after iTBS stimulation. In the study, their EEG responses were recorded while patients passively listened to isochronous speech sequences. The isochronous speech sequences included syllables, and four trisyllabic artificial words were constructed, adapted from the seminal study by Saffran et al. [15]. Organisms can extract statistical information from sequences of sounds, called statistical learning [15]. Statistical learning is a major theoretical construct in cognitive science.

Healthy adult language learning is antagonized by higher cognitive mechanisms, and inhibitory continuous theta burst stimulation of the left DLPFC improves adult language acquisition by unlocking implicit statistical learning mechanisms [28]. There is a competitive relationship between executive functions and statistical learning [17]. Mature cognitive mechanisms may constrain the implicit statistical learning mechanisms that contribute to early language acquisition. However, the effect of higher cognitive function on statistical learning in patients with severe brain injury has not yet been established. In the study, excitatory intermittent theta burst stimulation was used to induce long‐term potentiation in the left DLPFC, and an increasing trend in phrase‐level neural tracking was observed in the TMS group. The findings of this study indicated that the cognitive architecture in patients with severe brain injury unlocked statistical learning mechanisms that are likely to contribute to early language acquisition, thereby facilitating recovery of consciousness in these patients.

The study also had some limitations. The number of patients enrolled in this study is insufficient. The insufficient sample also affects the results of statistical analysis. The multi‐center studies for the efficacy of iTBS for prolonged disorders of consciousness should be conducted in the future with larger sample sizes. The heterogeneity of DOC etiology is also a limitation of the study. Further iTBS studies of DoC should be conducted based on etiology stratification.

This experimental study revealed that iTBS of the left DLPFC facilitated the recovery of consciousness in patients with DoC, and the underlying mechanisms involved may be through enhancing low‐frequency neural activity after iTBS treatment.

## Author Contributions

Benyan Luo contributed to the conception and design of the study; Chuan Xu, Jun Hu, Qianqian Wu, Jie Yu, Min Wu, Jian Gao, and Jingqi Li contributed to the acquisition and analysis of data; Chuan Xu and Qianqian Wu contributed to drafting the text and preparing the figures.

## Funding

This work was supported by the Science and Technology Innovation 2030 – “Brain Science and Brain‐like Research” Key Project (2022ZD0208905), the National Natural Science Foundation of China (U22A20293, 8250050433), the Natural Science Foundation of Zhejiang Province (QN25H090004, QN25H090042), the China Postdoctoral Science Foundation (2024M752878), and the Zhejiang Province Basic Public Welfare Research Program (LQ24H090006).

## Ethics Statement

The study was approved by the Ethical Committee of the Hangzhou Mingzhou Brain Rehabilitation Hospital. Written informed consent was provided by the participants or their legal guardians for the experiments and the publication of their individual details in this study.

## Consent

The authors have nothing to report.

## Conflicts of Interest

The authors declare no conflicts of interest.

## Data Availability

The data that support the findings of this study are available on request from the corresponding author. The data are not publicly available due to privacy or ethical restrictions. The preprocessed data supporting the results in the paper will be shared after the article is accepted.
